# Modifizierbare Risiko- und Schutzfaktoren für Demenz: Etablierte und neue Evidenz im Überblick

**DOI:** 10.1007/s00103-026-04279-7

**Published:** 2026-07-24

**Authors:** Felix G. Wittmann, Andrea E. Zülke, Michelle Vajnberger, Melanie Luppa, Steffi G. Riedel-Heller

**Affiliations:** Institut für Sozialmedizin, Arbeitsmedizin und Public Health, Medizinische Fakultät, Universität Leipzig, Philipp-Rosenthal-Straße 55, 04103 Leipzig, Deutschland

**Keywords:** Demenz, Risikoreduktion, Public Health, Intervention, Gesundheitspolitik, Dementia, Risk reduction, Public health, Health policy, Intervention studies

## Abstract

Die Alzheimer-Erkrankung und andere Formen von Demenz zählen zu den schwerwiegenden Volkskrankheiten, deren gesellschaftliche und gesundheitssystemische Bedeutung in den kommenden Jahren weiter zunehmen wird. Prognosen zur Krankheitslast sind entsprechend besorgniserregend. Gleichzeitig wurden in der Forschung, insbesondere im Bereich der Risikoreduktion, erhebliche Fortschritte erzielt. So ist eine Vielzahl etablierter Schutz- und Risikofaktoren auf individueller Ebene identifiziert worden und etliche daran anknüpfende Interventionsstudien wurden durchgeführt. Zunehmend richtet sich der Fokus jedoch auf bevölkerungsbezogene Maßnahmen zur langfristigen Reduktion der Demenzfälle.

Dieser Beitrag gibt einen Überblick über den aktuellen Forschungsstand. Ausgehend von der Datenlage zur Krankheitslast wird zunächst auf etablierte Risikofaktoren eingegangen. Darauf aufbauend werden neuere, bislang weniger berücksichtigte potenzielle Einflussfaktoren erläutert, bestehende Interventionsansätze zusammengefasst und die Bedeutung bevölkerungsbezogener Präventionsstrategien für Public Health und Gesundheitspolitik eingeordnet.

## Abstract (en)

Alzheimer’s disease and other dementias are among the most serious widespread diseases, with societal and healthcare-system relevance expected to increase further in the coming years. Projections of the future disease burden are correspondingly concerning. At the same time, considerable progress has been made in research, particularly in the field of risk reduction. A broad range of established protective and risk factors at the individual level has been identified, alongside a growing number of related intervention studies. Increasingly, however, attention is shifting toward population-based measures aimed at reducing dementia cases in the long term.

This article provides an overview of the current state of research. First, drawing on recent data on disease burden, established risk factors are summarized. Building on this, more recent and previously less considered potential determinants are discussed. The review further outlines existing intervention approaches and examines the relevance of population-based prevention strategies for public health and health policy.

## Einleitung

Demenzen zeichnen sich durch schwere kognitive Beeinträchtigungen aus, die die Fähigkeit einer Person, ihr Leben selbstständig zu bewältigen, im Verlauf der Erkrankung immer weiter einschränken. Das Risiko für Demenzen, etwa die Alzheimer-Erkrankung als häufigste Form, steigt mit dem Alter an. Aufgrund der Bevölkerungsalterung wird ein deutlicher Anstieg der Zahl von Menschen mit Demenz prognostiziert, die von weltweit ca. 55 Mio. (2019) auf bis zu 150 Mio. (2050) ansteigen wird. In Deutschland wird derzeit von etwa 1,8 Mio. Menschen mit Demenz ausgegangen; bis 2050 wird ein Anstieg auf bis zu 2,7 Mio. Personen erwartet [1].

Trotz Fortschritten in der Entwicklung kausaler Therapien, die in den Pathomechanismus der Erkrankung eingreifen [2, 3], sind Demenzen bislang nicht heilbar. Sie zeichnen sich zudem durch eine mitunter lange präklinische Phase aus, in der altersbedingte und pathologische Abbauprozesse ineinandergreifen. Daher kommt präventiven Maßnahmen – einschließlich Risikoreduktion (Primärprävention) sowie Früherkennung und rechtzeitiger Diagnose (Sekundärprävention) – große Bedeutung zu, um möglichst viele Fälle zu verhindern bzw. zu verzögern. International wurden in den vergangenen 2 Jahrzehnten enorme Forschungsanstrengungen unternommen, um potenziell beeinflussbare Risikofaktoren für Demenzerkrankungen zu identifizieren.

Die vorliegende Arbeit beschreibt den aktuellen Forschungsstand zu etablierten sowie weiteren aktuell diskutierten modifizierbaren Risikofaktoren für Demenzen. Systematische Übersichten und Metaanalysen, randomisiert kontrollierte Studien sowie epidemiologische Kohorten bilden hierfür die Grundlage. Dabei werden Faktoren auf individueller (Verhaltensprävention) sowie struktureller und Bevölkerungsebene (Verhältnisprävention) unterschieden und mögliche Implikationen für Präventionsansätze diskutiert.

## Potenziell modifizierbare Risiko- und Schutzfaktoren für Demenzen

### Etablierte Evidenz für verschiedene Risikofaktoren

Die „Lancet Commission on Dementia Prevention, Intervention and Care“ nennt in ihrem aktuellen Bericht insgesamt 14 potenziell beeinflussbare Risikofaktoren für Demenz. Diese umfassen geringe Bildung im frühen Lebensalter, Gehörverlust, erhöhtes LDL-Cholesterin („low-density lipoprotein“), Depression, Schädel-Hirn-Traumata, Bewegungsmangel, Diabetes mellitus, Rauchen, Bluthochdruck, Adipositas und übermäßigen Alkoholkonsum im mittleren Lebensalter sowie soziale Isolation, Exposition gegenüber Luftverschmutzung und unbehandelte Sehstörungen im höheren Lebensalter. Es wird davon ausgegangen, dass diese Faktoren zusammengenommen für bis zu 45 % aller Demenzfälle verantwortlich sind [4].

Zwar geht diese Schätzung von der unrealistischen Annahme aus, dass die genannten Faktoren vollständig eliminiert werden können. Dennoch verdeutlicht diese Zahl das erhebliche Potenzial, das der Modifikation von Risikofaktoren für die Prävention von Demenzen zukommt. Kohortenstudien aus Europa und den USA belegen einen Rückgang der altersspezifischen Inzidenz um 13 % pro Dekade über einen Zeitraum von 27 Jahren [5]. Auch in Deutschland konnte eine leicht sinkende Prävalenz beobachtet werden (1,2 Prozentpunkte pro Jahr zwischen 2009 und 2012; [1]). Weitere Studien, etwa aus Großbritannien, berichten ähnliche Kohorteneffekte mit geringerer altersüblicher Prävalenz von Demenzen bei später geborenen Personen [6]. Dies wird u. a. auf Veränderungen in modifizierbaren Risikofaktoren zurückgeführt, etwa gestiegene durchschnittliche Bildung oder Verbesserungen im Management kardiovaskulärer Erkrankungen.

Nach einer aktuellen Arbeit, basierend auf Daten des bevölkerungsrepräsentativen Deutschen Alters-Surveys, können in Deutschland 36 % aller Demenzfälle auf 12 modifizierbare Risikofaktoren zurückgeführt werden (geringe Bildung, Hörverlust, hoher LDL-Cholesterinspiegel, Depressionen, Bewegungsmangel, Rauchen, Diabetes, Bluthochdruck, Adipositas, hoher Alkoholkonsum, soziale Isolation, Sehbehinderung). Die prozentual stärkste Bedeutung kam dabei Depression, Hörverlust, geringer Bildung und Adipositas zu. Bei einer Reduktion aller genannten Risikofaktoren auf Bevölkerungsebene um 15–30 % bis 2050 wird – im Vergleich zu einer unveränderten Entwicklung ohne Intervention – von 170.000 bis 330.000 weniger Demenzfällen im Jahr 2050 ausgegangen [7].

### Neue Evidenz für weitere Risiko- und Schutzfaktoren

Zahlreiche neuere Studien belegen den Einfluss weiterer Faktoren, die dem individuellen Verhalten und Lebensstil zugeschrieben werden. Hierzu zählen Schutzfaktoren wie etwa verschiedene *Freizeitaktivitäten und geistig anregende Tätigkeiten* (bspw. Rätsel lösen, Spiele spielen [8], das Spielen eines Musikinstruments [9], kreative Tätigkeiten wie Malerei [10] oder der Besuch von Ausstellungen und Museen [11]). Dabei fanden einzelne Studien Dosis-Wirkungs-Effekte, so fiel etwa in einer japanischen Studie das Demenzrisiko umso geringer aus, je mehr Hobbys Personen nachgingen [12]. Auch scheinen aktive Freizeitaktivitäten eine größere protektive Wirkung aufzuweisen als passive Tätigkeiten wie etwa Fernsehen [8]. Weitere Untersuchungen müssen klären, welche Bedeutung jeweils verschiedenen Komponenten dieser Aktivitäten zukommt, bspw. der kognitiven Beanspruchung durch die ausgeübte Tätigkeit, aber auch den körperlichen Anforderungen und dem sozialen Austausch, der mit vielen Freizeitaktivitäten einhergeht.

Bereits seit Längerem werden Zusammenhänge zwischen *Ernährung* und kognitiver Leistung sowie Demenz untersucht. Entsprechende Arbeiten betrachten dabei sowohl den Konsum einzelner Lebensmittel, beispielsweise den Verzehr von Obst und Gemüse oder den Konsum hochverarbeiteter Lebensmittel, als auch gesamte Ernährungsstile. Dazu gehören die mediterrane Ernährung und darauf basierende Ernährungsformen (bspw. die Dietary-Approaches-to-Stop-Hypertension-(DASH-)Diät oder Mediterranean-DASH Intervention for Neurodegenerative Delay (MIND)). Während Metaanalysen prospektiver Kohortenstudien positive Zusammenhänge zwischen der mediterranen Ernährung (hoher Obst- und Gemüsekonsum, pflanzliche Fette, hoher Konsum von fettreichem Fisch, Hülsenfrüchte, geringer Konsum von rotem Fleisch) und kognitiver Leistung konstatieren [13], ist die Evidenz aus Interventionsstudien gemischt. Übersichtsarbeiten zu randomisiert kontrollierten Studien konnten zwar Effekte der mediterranen oder DASH-Ernährung auf die kognitive Leistung in einzelnen Domänen, jedoch nur vereinzelt auf das Gesamtrisiko für Demenz feststellen [14–16].

Metaanalysen ergaben verringerte Risiken für kognitiven Abbau und Demenz bei höherem Obst- und Gemüsekonsum [17, 18]. Dagegen nimmt die Evidenz für Zusammenhänge zwischen einem höheren Verzehr hochverarbeiteter Lebensmittel wie Softdrinks, abgepackten Snacks und Backwaren sowie Fertiggerichten und einem gesteigerten Demenzrisiko zu [19, 20]. Dies könnte unter anderem durch deren hohen Gehalt an Kalorien, Zucker und ungesättigten Fettsäuren bedingt sein [21].

Die wissenschaftliche Evidenz zu Zusammenhängen zwischen Fleischkonsum und Demenzrisiko ist bislang uneinheitlich, was insbesondere auf die hohe Heterogenität der zugrunde liegenden Studien zurückgeführt werden könnte [22]. Positive Effekte werden moderatem Kaffeekonsum (3–5 Tassen pro Tag) zugeschrieben. Dies geht u. a. aus einer Metaanalyse hervor, die einen u‑förmigen Zusammenhang zwischen Kaffeekonsum und dem Risiko für Alzheimer-Demenz berichtet [23]. Die Evidenz zu Zusammenhängen zwischen Ernährung und dem Risiko für Demenzen ist derzeit jedoch noch begrenzt. Heterogenität in der Erfassung von Nahrungsmittelkonsum, Beobachtungszeitraum und weiteren Faktoren, die mit Ernährung zusammenhängen, schränken die Verallgemeinerbarkeit der Ergebnisse ein.

Wissenschaftliche Erkenntnisse mehren sich hingegen hinsichtlich *Emotionen und psychischem Wohlbefinden* als Einflussfaktoren für Kognition und Demenz. In Studien aus den USA, Südkorea und Japan waren etwa positiver Affekt und höhere Lebenszufriedenheit mit verlangsamtem kognitiven Abbau [24, 25] und geringerem Demenzrisiko [26] verbunden. Ein Lebensziel zu haben war in 2 Metaanalysen mit einem um über 20 % verringerten Demenzrisiko assoziiert [27, 28], wohingegen keine Assoziationen mit berichtetem Glück belegt sind [29, 30]. Derartige Befunde könnten u. a. durch negative emotionale Zustände erklärbar sein, die zumeist nicht simultan untersucht wurden und zwischen beobachteten Personen variieren können. Einzelne Arbeiten legen positive Effekte von Meditation und Achtsamkeitsübungen (bspw. Yoga) nahe, die sich positiv auf das emotionale Wohlbefinden auswirken können [31–33]. Eine systematische Übersichtsarbeit belegt darüber hinaus Assoziationen zwischen psychischem Stress und erhöhtem Demenzrisiko [34].

Während Depressionen bereits zu den etablierten Risikofaktoren für Demenzen zählen, liefern neuere Studien Hinweise auf negative Effekte von weiteren *psychischen Erkrankungen*. So sind laut Metaanalysen sowohl bipolare Störungen als auch diagnostizierte Angststörungen mit einem erhöhten Demenzrisiko verbunden [35, 36]. Für posttraumatische Belastungsstörungen (PTBS) fand sich in einzelnen Studien ein erhöhtes Risiko [37], während andere keine signifikanten Zusammenhänge berichteten [36]. Zudem gibt es Hinweise auf erhöhte Demenzrisiken bei Personen mit psychotischen Störungen [37, 38]. Indes können sich Symptome von Psychosen und Demenz überschneiden; es kann nicht ausgeschlossen werden, dass ältere Teilnehmende in den entsprechenden Studien ggf. bereits erste Demenzsymptome aufwiesen. Insgesamt deutet die Studienlage auf negative Effekte verschiedener psychischer Störungen hin, jedoch müssen weitere Untersuchungen diese ersten Befunde erhärten.

Schließlich gibt es zunehmend Evidenz zu *Schlafproblemen* als Risikofaktor für Demenz, wobei bisherige Studien sowohl objektiv gemessenen als auch subjektiv wahrgenommenen Schlaf berücksichtigen [39]. Eine niederländische Studie legt nahe, dass übermäßige Bettliegezeit (≥ 9 h) sowie häufige nächtliche Schlafunterbrechungen mit verschlechterter Exekutivfunktion und Aufmerksamkeit sowie geringerer grauer Substanz im Gehirn verbunden sind [40]. Tab. 1 gibt einen Überblick über etablierte und aktuell diskutierte Risikofaktoren für Demenzen auf individueller Ebene (Mikroebene).

**Tab. 1:** Lebensstil- und gesundheitsbezogene Risikofaktoren für Demenzen

Risikofaktor	Definition/Erklärung	Evidenzgrad
Geringer Bildungsstand	RR für Demenz: 1,6 für Personen mit niedriger vs. hoher Bildung [4]	+++
Hörverlust	RR für Demenz: 1,4 pro 10 dB Hörverlust [4]	+++
Bluthochdruck	RR für Demenz: 1,2 für Personen mit vs. ohne Hypertonie [4]	+++
Rauchen	RR für Demenz: 1,3 für Raucher*innen vs. Nicht‑/Ex-Raucher*innen [4]	+++
Adipositas	RR für Demenz: 1,3 für Personen mit vs. ohne Adipositas (BMI >30 kg/m^2^; [4])	+++
Depression	RR für Demenz: 2,2 für Personen mit vs. ohne Depression [4]	+++
Bewegungsmangel	RR für Demenz: 1,2 für körperlich inaktive vs. aktive Personen [4]	+++
Diabetes mellitus	RR für Demenz: 1,7 für Personen mit vs. ohne Diabetes mellitus [4]	+++
Erhöhtes LDL-Cholesterin	RR für Demenz: 1,3 für Personen mit LDL-Cholesterin >3 mmol/l vs. Personen mit niedrigerem LDL-Cholesterin [4]	++
Übermäßiger Alkoholkonsum	RR für Demenz: 1,2 bei Konsum von ≥168 g Ethanol/Woche vs. < 168 g Ethanol/Woche [4]	+++
Schädel-Hirn-Traumata	RR für Demenz: 1,7 für Personen mit vs. ohne traumatische Schädel-Hirn-Verletzungen in der Vergangenheit [4]	+++
Soziale Isolation	RR für Demenz: 1,6 für sozial isolierte Personen vs. sozial integrierte Personen [4]	+++
Sehverlust	RR für Demenz: 1,5 bei Personen mit vs. unbehandeltem Sehverlust [4]	++
Schlafprobleme	u. a. Insomnie, Schlaffragmentierung, verlängerte Einschlaflatenz, REM-Schlaf-Verhaltensstörung, übermäßige Bettliegezeit Schlafapnoe, erhöhte Schlafdauer (mit erhöhtem Demenzrisiko assoziiert; [39])	++
Freizeitaktivitäten	Positive Effekte von geistig anregenden Tätigkeiten (Lesen, Kreuzworträtsel, Gruppenaktivitäten, Besuch kulturelle/gesellschaftliche Veranstaltungen) auf kognitive Leistungsfähigkeit und Demenzrisiko [41, 42]	+
Ernährung	Mediterrane Ernährung mit geringerem Risiko für Demenz assoziiert (RR: 0,89; [13])	++
Psychisches Wohlbefinden	Höherer positiver Affekt (HR = 0,75) und Sinn im Leben (HR = 0,76; [25]) mit geringerem Demenzrisiko assoziiert	+
Psychische Erkrankungen	Bipolare Störung (pooled OR = 2,23; [35]) sowie Angststörung/-zustände (pooled OR = 1,60; [36]) mit erhöhtem Demenzrisiko assoziiert	+

*HR* „hazard ratio“ (Risikoquotient); *LDL* „low-density lipoprotein“; *RR* „risk ratio“ (relatives Risiko); *dB* Dezibel; *BMI* Body-Mass-Index; *OR* „odds ratio“ (Chancenverhältnis; *pooled OR* zusammengefasste Effektgröße aus Metaanalyse) + neue Erkenntnisse, erste Evidenz; ++ solide Evidenz; +++ Metaanalysen

Neben den beschriebenen Risikofaktoren, die im weitesten Sinne dem individuellen Verhalten und Lebensstil zugeschrieben werden, beeinflussen auch Faktoren auf Bevölkerungsebene (Meso- und Makroebene) das Risiko für kognitiven Abbau und Demenzerkrankungen (Tab. 2). Hierzu zählen etwa Merkmale des *Wohnumfelds* wie soziodemografische Zusammensetzung, Wohndichte, Zugang zu Versorgung sowie soziale und kulturelle Angeboten oder das Verhältnis von Wohn- und Grünflächen. Zusammenhänge von Feinstoffbelastungen und erhöhtem Demenzrisiko sind gut belegt [4]. Doch auch die Exposition gegenüber Lärm, beispielsweise durch Verkehr, kann sich negativ auf die kognitive Leistungsfähigkeit und das Risiko für Demenzen auswirken [43]. Eine Metaanalyse berichtete negative Zusammenhänge zwischen einem ländlichen Wohnumfeld bzw. Deprivation auf Wohnortebene und dem Demenzrisiko, während ein besserer Zugang zu Grünflächen mit einem verringerten Risiko assoziiert war [44]. Zudem finden sich Hinweise darauf, dass ein höherer Urbanisierungsgrad sowie eine höhere Wohndichte mit einem erhöhten Demenzrisiko verbunden sind [45].

**Tab. 2:** Risikofaktoren für Demenzen auf Bevölkerungsebene

Risikofaktor	Definition/Erklärung	Evidenzgrad
Materielle Ressourcen	Höheres Einkommen [64] und Vermögen (Sach‑, Geschäfts- und Finanzvermögen; [69]) mit geringerem Demenzrisiko assoziiert	++
Luftverschmutzung	RR für Demenz: 1,1 für jede Einheit Zunahme von Feinstaubpartikeln mit Durchmesser von ≤ 2,5 μm und ≤ 10 μm [4]	++
Weitere Merkmale des Wohnumfelds	Zunahme von Grünflächen in der Wohnumgebung mit langsamerem kognitiven Abbau assoziiert (b = 0,02; [71]); Straßenverkehrs- und Eisenbahnlärm mit höherem Demenzrisiko assoziiert (HR: je 1,16); höhere Wohndichte und Urbanität mit höherem Demenzrisiko assoziiert [45]	++
Merkmale des Arbeitsumfelds	Arbeitsbedingter Stress (OR = 1,53; [72]) sowie Nachtschichtarbeit (HR = 1,47; [62]) mit höherem Demenzrisiko assoziiert; höhere Arbeitsbelastung bei Renteneintritt mit geringerer kognitiver Leitungsfähigkeit assoziiert (Schätzung: −1,33; [61]); geringe kognitive Anforderungen am Arbeitsplatz mit erhöhtem Demenzrisiko assoziiert (RR = 1,31; [65])	+

*RR* „risk ratio“ (relatives Risiko); *OR* „odds ratio“ (Chancenverhältnis); *μm* Mikrometer; *b* Koeffizient; + neue Erkenntnisse, erste Evidenz; ++ solide Evidenz; +++ Metaanalysen

Städtische Wohnumgebungen können sich jedoch auch positiv auf Kognition und Demenzrisiko auswirken, etwa durch bessere Erreichbarkeit von Versorgungsstrukturen, öffentlichem Nahverkehr und Einrichtungen des täglichen Bedarfs [46]. Ebenso belegt eine Übersichtsarbeit Zusammenhänge u. a. zwischen Bevölkerungsdichte, höherer Straßenvernetzung, besserer Begehbarkeit, mehr Ressourcen innerhalb der Nachbarschaft (etwa Gemeindezentren, Supermärkte und Bibliotheken) und höherer Qualität der bebauten Umgebung (z. B. Verkehrssicherheit oder Sauberkeit) mit besserer kognitiver Leistungsfähigkeit und verringertem Demenzrisiko [47]. Während Leben in einer sozial benachteiligten Nachbarschaft in mehreren Studien mit stärkerem kognitiven Abbau und höherem Demenzrisiko verbunden war [48–50], waren Zusammenhänge unter Kontrolle individueller soziodemografischer Merkmale mitunter nicht mehr signifikant [51].

Die komplexen Zusammenhänge von Umgebungsmerkmalen mit Demenzen sind noch nicht vollständig untersucht und nicht alle Assoziationen folgen einem linearen Verlauf. So findet sich bspw. der höchste Anteil an Grünflächen oft in ländlichen Gebieten, in denen wiederum der Zugang zu Versorgungsstrukturen und Einrichtungen des täglichen Bedarfs erschwert sein kann [52].

Auch spezifische Merkmale des *Arbeitsumfelds* stehen mit dem Demenzrisiko in Verbindung, so existieren bspw. Belege für den schädlichen Einfluss der Exposition gegenüber Magnetfeldern [53] sowie Pestiziden [54] am Arbeitsplatz auf Kognition und Demenzrisiko. Erhöhter Stress im Job sowie hohe Anforderungen bei geringen Kontrollmöglichkeiten stehen mit schnellerem kognitiven Abbau in Verbindung [55, 56]. Ebenso zeigen sich erhöhte Demenzrisiken bei regelmäßiger Nacht- und unregelmäßiger Schichtarbeit [57]. Dagegen zeigen zahlreiche Studien, dass Arbeitstätigkeiten mit hohen kognitiven Anforderungen mit geringerem Demenzrisiko verbunden sind [58–60].

Zusammenhänge zwischen *materiellen Ressourcen* und dem Risiko für Demenzen sind ebenfalls gut belegt. So war ein höheres Einkommen in US-amerikanischen Studien mit besserer kognitiver Leistung bei älteren Erwachsenen assoziiert [61, 62] und erklärte bis zu 12 % der Leistungsunterschiede zwischen Teilnehmenden, die als „schwarz“ bzw. „weiß“ klassifiziert wurden [63]. Weitere internationale Studien belegen geringere Demenzrisiken bei höherem Einkommen, während Armut sowie hohe finanzielle Verluste mit höheren Risiken assoziiert waren [64, 65]. Auch relative Maßstäbe für fehlende materielle Ressourcen, wie bspw. Krankenversicherung oder relatives Einkommen, stehen im Zusammenhang mit erhöhtem Demenzrisiko [66–68]. Materielle Einflüsse, wie etwa geringes Einkommen, können hierbei auch indirekte Effekte aufweisen, etwa durch negative Effekte ungünstiger Wohnumgebungen (s. oben), oder auch in erschwertem Zugang zu gesundheitsförderlichen Verhaltensweisen. So waren in einigen Studien Unterschiede in kognitiver Leistung zwischen sozialen Statusgruppen zumindest in Teilen durch Lebensstilfaktoren erklärbar [69, 70].

## Interventionsstudien

In Anbetracht der Vielzahl modifizierbarer Risikofaktoren wurden in den letzten Jahren zahlreiche Interventionsstudien initiiert, die durch eine Verbesserung von bspw. Ernährungs- und Bewegungsverhalten sowie Management kardiovaskulärer und metabolischer Risikofaktoren auf den Erhalt kognitiver Leistung im Alter abzielen. Wegweisend hierfür war die Finnish Geriatric Intervention Study to Prevent Cognitive Impairment and Disability (FINGER), in der 1152 ältere Erwachsene (60–77 Jahre) mit erhöhtem Demenzrisiko entweder eine multimodale Intervention (Optimierung von Ernährung, Bewegung und sozialen Aktivitäten, kognitives Training sowie Management kardiovaskulärer Risikofaktoren) oder als Kontrollgruppe die reguläre Gesundheitsversorgung erhielten. Die Intervention verbesserte in einem Zeitraum von 2 Jahren sowohl die kognitive Leistungsfähigkeit als auch weitere sekundäre Zielgrößen [73].

Basierend auf diesen Ergebnissen wurde 2017 das World-Wide-FINGERS-(WW-FINGERS-)Netzwerk ins Leben gerufen, um das Konzept von FINGER an unterschiedliche geografische, kulturelle und ökonomische Kontexte anzupassen, zu testen und zu harmonisieren [74]. Derzeit (Stand: Februar 2026) existieren entsprechende Studien in unterschiedlichen Stadien in 73 Ländern weltweit. Die Ergebnisse abgeschlossener Studien sind gemischt: So zeigten einige Interventionen positive Auswirkungen auf die kognitive Leistungsfähigkeit, während andere keine signifikanten Effekte berichten [75]. Hervorzuheben sind u. a. die US-amerikanische U.S.-POINTER-Studie aufgrund der großen, landesweit rekrutierten Stichprobe und eines translationalen Ansatzes in einer realweltlichen Population. Diese konnte positive Effekte der Multikomponenten-Intervention auf die kognitive Leistung nachweisen [76]. Auch der südkoreanische SUPERBRAIN-Trial, durchgeführt bei Teilnehmenden mit leichter kognitiver Störung, berichtete positive Effekte einer Multikomponenten-Intervention auf kognitive Leistungsfähigkeit [77], während die japanische J‑MINT-Studie, die ebenfalls Personen mit leichten kognitiven Beeinträchtigungen untersuchte, keine Effekte auf kognitive Endpunkte verzeichnete. Diese neueren Studien zeichnen sich v. a. durch die Integration moderner Biomarker und Bildgebung sowie den Fokus auf praktische Implementierung im Gesundheitssystem aus [78].

In Deutschland war der AgeWell.de-Trial die erste randomisierte kontrollierte Studie, die die Wirksamkeit einer auf FINGER basierenden Intervention auf die kognitive Leistungsfähigkeit und mehrere sekundäre Endpunkte bei älteren Hausarztpatient*innen mit erhöhtem Demenzrisiko untersuchte. Über Hausarztpraxen an 5 Studienzentren (Leipzig, Halle, München, Kiel und Greifswald) wurden 1030 ältere Personen (60–77 Jahre) in die Studie eingeschlossen und erhielten entweder eine multimodale Lebensstilintervention (Ernährungsberatung, Förderung der körperlichen, kognitiven und sozialen Aktivitäten, Optimierung der Medikation sowie – bei Bedarf – gezielte Maßnahmen bei Trauer und depressiven Symptomen) oder allgemeine Gesundheitsinformationen zu den Interventionskomponenten. Primäres Ziel war der Erhalt kognitiver Leistungsfähigkeit über 2 Jahre; sekundär wurden Lebensqualität, soziale Isolation, depressive Symptomatik und Kosteneffektivität erfasst [79].

Die AgeWell.de-Studie konnte zeigen, dass eine pragmatische, in die Primärversorgung integrierte Lebensstilintervention das Demenzrisiko in einer Risikopopulation signifikant senkt. Dies zeigen Analysen mit dem LIBRA-Index (LIfestyle for BRAin Health), welcher das Risiko basierend auf mehreren Risiko- und Schutzfaktoren misst [80]. Die Studie leistet damit einen wichtigen Beitrag zur Evidenzbasis modifizierbarer Lebensstilfaktoren in der Demenzprävention und stellt einen bedeutenden Schritt zur Implementierung multidimensionaler Präventionssätze dar.

## Prävention auf Bevölkerungsebene – worauf es ankommt

Bisherige Maßnahmen der Demenz-Risikoreduktion konzentrierten sich überwiegend auf Maßnahmen auf individueller Ebene [81]. Für eine nachhaltige Reduktion des Demenzrisikos in der Gesamtbevölkerung, eine spürbare Senkung der Krankheitslast und eine Verringerung sozialer Ungleichheiten sind jedoch bevölkerungsbezogene Ansätze erforderlich [82]. Solche Maßnahmen wirken potenziell langfristig, da sie Risiken frühzeitig und dauerhaft senken („Legacy-Effekt“). Ihre positiven Effekte akkumulieren sich im Verlauf des Lebens, wodurch sie mit der Zeit wirksamer und kosteneffizienter werden, auch ohne wiederholte Umsetzung oder zusätzliche Finanzierung [81].

Die Risikoreduktion auf Bevölkerungsebene zielt darauf ab, gesellschaftliche Rahmenbedingungen so zu gestalten, dass weniger Faktoren entstehen, die Demenz begünstigen, und gleichzeitig Faktoren gefördert werden, die vor einer Demenzerkrankung schützen. Im Zentrum stehen strukturelle sowie politische Veränderungen, die gesundheitsförderliches Verhalten zugänglich machen sollen. Diese Maßnahmen sollten auf allen politischen Ebenen ansetzen, einzelne wie kombinierte Risikofaktoren adressieren und soziale Ungleichheiten in der Bevölkerung abbauen [81, 83].

Typische bevölkerungsbezogene Maßnahmen umfassen erstens politische und strukturelle Interventionen zur Reduktion einzelner veränderbarer Risikofaktoren, etwa durch Tabakkontrollgesetze. Zweitens zielen sie auf Veränderungen der Lebensumwelt ab, die mehrere Risikofaktoren gleichzeitig beeinflussen, beispielsweise durch bewegungsfördernde Umgebungen oder einen verbesserten Zugang zu gesunder Ernährung. Drittens adressieren sie grundlegende gesellschaftliche Ursachen von Demenzrisiken, darunter Armut, eingeschränkte soziale Teilhabe sowie strukturelle Faktoren wie Diskriminierung, die indirekt über ungleiche Lebensbedingungen und Gesundheitschancen wirken. Ziel ist ein ganzheitlicher Ansatz, der die sozialen, wirtschaftlichen und politischen Rahmenbedingungen verbessert, welche das Demenzrisiko langfristig prägen [81]. Einige dieser Strategien sind aus der Prävention nichtübertragbarer Krankheiten bekannt, andere speziell für Demenz relevant [83]. Ein bedeutender Mehrwert bevölkerungsbezogener Maßnahmen ist die Möglichkeit sogenannter *Overall-Health-*Verbesserungen, die über die Reduktion des Demenzrisikos hinaus vielfältige gesundheitsfördernde Effekte haben.

Konkrete Beispiele umfassen eine alternsgerechte Stadtplanung, die Förderung sozialen Zusammenhalts, Investitionen in Bildung, rauchfreie Innenräume, die Regulierung von Salz- und Zuckergehalt in Lebensmitteln oder der Ausbau hochwertiger Grünflächen [82]. Diese Maßnahmen zielen darauf ab, Risikofaktoren für Demenz wie Rauchen, geringe Bildung, Bluthochdruck, Übergewicht, Depression und soziale Isolation zu verringern und Schutzfaktoren zu stärken. Im Gegensatz zu individualmedizinischen Ansätzen erfordern sie das Zusammenwirken vieler Akteur*innen und sind daher häufig politisch geprägt [82].

Bevölkerungsweite Maßnahmen sind jedoch i. d. R. schwieriger umzusetzen als individuelle Interventionen, da sie kurzfristig Widerstand hervorrufen oder politisch unpopulär sein können, während ihr langfristiger Nutzen oft unterschätzt wird. Zudem reichen klassische Forschungsdesigns wie klinische Studien häufig nicht aus, um ihre Wirksamkeit nachzuweisen. Ihre Umsetzung erfordert sektorübergreifende Koordination und langfristiges, systemisches Denken [81].

## Die Gefahr der Stigmatisierung

Während die Erkenntnisse zu Potenzialen der Risikoreduktion von Demenz Grund zur Hoffnung bieten, bergen sie, nicht zuletzt durch die stark auf das Individuum fokussierenden Ansätze, zugleich Risiken von Stigmatisierung. Schließlich lassen sich nicht alle Risikofaktoren gleichermaßen beeinflussen und lassen sich aus statistischen Zusammenhängen keine Schlüsse auf einzelne Personen ableiten: Auch durch einen gesunden Lebensstil ist das Risiko für Demenz nicht vollständig beeinflussbar. Übermäßige Fokussierung auf individuelle Maßnahmen der Risikominimierung, etwa durch gesundheitsförderliches Verhalten, birgt das Risiko, dass Personen für eine Demenzerkrankung in die Verantwortung genommen werden, indem die Erkrankung einseitig auf Lebensstil und Verhaltensweisen zurückgeführt wird. Maßgeblich zu nennen sind für eine große Bevölkerungsgruppe erhebliche individuelle und sozioökonomische Barrieren hinsichtlich präventiver Verhaltensweisen [84].

Dies lässt 2 Schlussfolgerungen zu. Zunächst sollte Prävention nicht ausschließlich auf der individuellen Ebene ansetzen. Es ist notwendig, inklusiv wirksame Interventionsstrategien mitzudenken, was erfordert, soziale Ungleichheit und strukturelle Hürden zu berücksichtigen. Zudem sollte Demenzprävention stärker positiv gerahmt werden, etwa durch die Betonung der Förderung psychischen Wohlbefindens. Im internationalen Kontext ist vermehrt von „Positive Brain Health“ die Rede, also positiver Hirngesundheit, statt von Demenz oder kognitivem Abbau. Schließlich tragen Risikofaktoren nur bedingt dazu bei, das individuelle Erkrankungsrisiko zu determinieren, und gute Hirngesundheit ist in allen Lebensphasen zentral für individuelles Wohlbefinden sowie für gesellschaftlichen Fortschritt [85].

## Fazit

Die Evidenz zu potenziell modifizierbaren Risikofaktoren für Demenz hat in den vergangenen Jahren enorme Fortschritte erzielt und entwickelt sich mit hohem Tempo weiter. Bestimmte Faktoren auf individueller Ebene, etwa lebensstilbezogene, kardiovaskuläre und psychosoziale Faktoren, gelten als modifizierbar. Darauf aufbauend zeigen Interventionsstudien erste Erfolge durch Lebensstilveränderungen. Gleichzeitig rückt der Fokus zunehmend auf bevölkerungsbezogene Maßnahmen. Dazu zählen beispielsweise die stärkere Berücksichtigung sozialer Ungleichheit, der Einfluss der Wohngegend oder der Zugang zu Gesundheitsversorgung, aber auch zu sozialen und kulturellen Angeboten. Während einzelne Personen durchaus an evidenten Risikofaktoren ansetzen können, ist dies nicht in gleichem Maße und nur bedingt für alle Mitglieder einer Gesellschaft möglich. Es ist daher von hoher Relevanz, dass hinsichtlich Risikoreduzierung strukturelle und politische Entscheidungen und Maßnahmen mitdiskutiert werden.

## Data Availability

Für die Studie wurden keine Datensätze erstellt oder analysiert. Eine Angabe zur Datenverfügbarkeit ist deshalb nicht notwendig.
